# Quality of Life among Obstetric Fistula Patients at Kitovu Mission Hospital: A Health Facility-Based Cross-Sectional Study in Masaka District, Uganda

**DOI:** 10.1155/2020/7953915

**Published:** 2020-05-20

**Authors:** Samuel Kakembo, Christine Atuhairwe, Ivan Mugisha Taremwa

**Affiliations:** Clarke International University, P.O. Box 7782, Kampala, Uganda

## Abstract

**Background:**

Obstetric fistula (OF) remains a silent neglected maternal challenge associated with devastating life consequences. Living with OF presents far-reaching physical, social, psychosocial, and emotional concerns, which negatively impact a woman's quality of life. This study evaluated the quality of life among obstetric fistula patients in Masaka district, Uganda.

**Method:**

A cross-sectional study was conducted among 63 women diagnosed with OF at Kitovu Mission Hospital. Data were collected using a questionnaire, observation, in-depth interviews, and focus group discussions. Data were analyzed at univariate, bivariate, and multivariate levels, where the ordinal logistic regression model was applied. The qualitative data was transcribed and analyzed using qualitative content analysis.

**Results:**

Majority (87%) of the women diagnosed with OF reportedly had a poor quality of life. Bivariate analysis indicated that level of education (*P* < 0.001), employment status (*P* < 0.001), energy for everyday life (*P* < 0.001), capacity to work (*P* < 0.001), satisfaction with personal relationships (*P* < 0.001), feelings of loneliness (*P* < 0.001), negative feelings (*P* = 0.002), and self-confidence (*P* < 0.001) were significantly associated with good QoL. Multivariate analysis showed increased odds of good QoL increased among women with self-confidence (OR = 32.320; CI = 2.019–517.467), formal education (OR = 9.9497; CI = 1.075–92.048), women who did not experience difficulties in mobility (OR = 19.144; CI = 0.149–2456.770), and women who were satisfied with their personal relationships (OR = 5.785; CI = 0.447–74.824).

**Conclusion:**

A holistic fistula treatment approach is required that takes into consideration all aspects of life to address the consequences of obstetric fistula to realize improved quality of life among patients.

## 1. Background

Birth injuries present a chain reaction of untold physical, social, and psychological pain and suffering in millions of women [1]. One such devastating complication is the obstetric fistula (OF) [2]. Global estimates show that at least 3 million women in poor countries have unrepaired vesicovaginal fistulas; of these, 30,000–130,000 new cases occur in Africa annually [3, 4]. Although preventable, the persistence of OF is a sign of global inequality and an indication that health systems are failing to protect the poorest and most vulnerable women [5]. The majority of OFs are due to obstructed labour, and expectedly, they are most prevalent in areas where maternal mortality is due to obstructed labour as a cause of maternal deaths [4, 5].

According to the Uganda Demographic and Health Survey (2016), 2% of Ugandan women aged 15–49 had experienced OF, and 368 women per 100,000 live births died of birth-related causes [6]. Also, for every death, about six individuals survive with chronic and debilitating ill health [7]. OF has several aftermaths on QoL, as some studies have indicated it is a distressing and disabling condition affecting social, psychological, occupational, domestic, physical, and sexual lives of 15–30% of women [8, 9]. Patients give up many aspects of their usual life with obvious detriment to their social interactions, interpersonal and sexual relationships, careers, and psychological wellbeing [4, 10]. Living with OF is associated with the experience of multiple losses, which negatively impact a woman's identity and QoL [11, 12]. Women living with OF are often limited by the existing coping options and they face numerous challenges [13, 14]. Also, they may lack adequate support and appropriate attention, making it a big concern [15, 16]. This is further complicated by the fact that many women living with OF are still unaware of the availability of its treatment and management [17, 18]. Consequently, an estimated 80% of these women do not seek treatment and live with the condition for several years [19]. As a result, this leads to chronic incontinence resulting in physical ailments such as frequent infections, kidney disease, painful sores, and infertility [5]. These present numerous psychosocial challenges like living socially deprived and isolated, living psychologically stigmatized and depressed, and living marital and sexual lives that are no longer joyful [5]. In this state, women either isolate themselves or are isolated by society and their close relatives, which consequently affects their QoL [4]. This study evaluated the QoL among obstetric fistula patients in Masaka district, Uganda.

## 2. Methods

### 2.1. Study Area

The study was carried at Kitovu Mission Hospital, located within Masaka Municipality in Masaka district in Uganda. The area of focus (Masaka district) was chosen because it bears Kitovu Mission Hospital which admits and treats over 300 obstetric fistula cases annually. Founded in 1955, Kitovu Mission Hospital is a 248-bed capacity Private Not for Profit (PNFP) Hospital, operating under the umbrella organization of the Uganda Catholic Medical Bureau (UCMB) (Kitovu-hospital.org, 2020). It has a specialized fistula repair clinic and it serves as a referral site for complex fistula repairs. The hospital is also used as a regular camping site for visiting master surgeons where many women with different fistula cases are attended to.

### 2.2. Study Design and Duration

The study used a cross-sectional study design conducted between June and October 2019.

### 2.3. Study Population and Recruitment

The study comprised women of child-bearing age (15–49 years) living with OF who consented. The study excluded all women who were too ill to be interviewed.

### 2.4. Sample Size Estimation

A census was used to obtain the number of women diagnosed with OF, since it is a rare condition. At the time of the study, all 63 women diagnosed with OF at Kitovu Mission Hospital were included in the study. These women participated in face-to-face key informant interviews and were equally divided into 7 FGDs consisting of 9 women per group.

### 2.5. Data Collection

The study utilized both quantitative and qualitative tools to obtain data to give insights into the experiences and perceptions of women living with OF, and the cumulative effects this malady has had on their QoL.

For the quantitative approach, the study adopted a modified version of the World Health Organization Quality of Life (WHOQoL-BREF) questionnaire. This consisted of 26 questions, 24 of which were divided into four domains: physical health, psychological health, social relationships, and environment. Slight additions were made to the tool to include patient demographic characteristics. For the standard questions, each item was rated on a 1–5-point Likert scale, where 1 indicated low (negative perception) and 5 indicated high (positive perception). The domain scores were computed by summing the items in each grouping in the respective domain, then dividing it by the number of aspects in each domain, after it was multiplied by 4. This was to enable comparison with the WHOQoL scores. The scores ranged from 4 to 20, with 4 being the worst and 20 the best.

### 2.6. Study Variables

The dependent variable QoL formed four latent constructs (physical health, psychological health, social relationships, and environment). For each latent construct, three categorical responses were generated (good, fair, and poor). These responses were summed up for each OF patient to generate corresponding categorical responses for QoL. All the 63 women participated in face-to-face key informant interviews aided by a prior designed quantitative questionnaire. For the qualitative data collection, these women were equally divided into 7 FGDs each consisting of 9 women and interviewed as aided by the focus group interview guide.

### 2.7. Data Management and Analysis

Data were analyzed at univariate, bivariate, and multivariate levels using SPSS version 20. At univariate level, a basic description of the respondents was done. At bivariate level, chi-square tests were performed to examine the association between the dependent and independent variables. At multivariate level, ordinal logistic regression model was used to establish the association between the independent variables which were significantly associated with quality of life. The qualitative data was transcribed and analyzed using qualitative content analysis.

## 3. Results

### 3.1. Sociodemographic Characteristics of Women with Obstetric Fistula

Sixty-three women living with OF were enrolled. Their mean age was 26.6 (range: 15–40) years. Majority (71%) of women reportedly had their first pregnancy when they were less than 20 years. Further, 46% of women had lived with OF for between 1 and 12 months, and majority (70%) experienced still births preceding fistula. These are summarized in Table 1.

### 3.2. Perceived QoL

Majority (87%) of women living with OF reported “poor,” 8% reported “fair,” and only 5% reported “good” QoL.

### 3.3. Association between Patients' Sociodemographic Factors and Their QOL

It was established that age of the respondents, marital status after OF, type of incontinence, and duration with OF were not significantly associated with QoL. Majority of the women with no education had poor QoL (95%; *P* < 0.001), all women not employed had poor QoL (100%; *P* < 0.001), and the highest proportion of women that had their first pregnancy below 20 had poorer QoL (91%; *P* < 0.001). Furthermore, QoL was significantly associated with outcome of delivery preceding OF (90%; *P* < 0.001), as given in Table 2.

### 3.4. Association between Physiological Determinants and Perceived QoL

The determinants were physiological or physical pain (*X*^2^ = 9.809; *P*=0.044), dependency on medical treatment to function in daily life (*X*^2^ = 16.777; *P*=0.010), difficulties in mobility (*X*^2^ = 7.764; *P*=0.022), energy for everyday life (*X*^2^ = 22.480; *P* < 0.001), satisfaction with sleep pattern (*X*^2^ = 6.439; *p*=0.040), ability to perform daily activities (*X*^2^ = 6.439; *p*=0.040), and capacity for work (*X*^2^ = 43.319; *p* < 0.001). Furthermore, all women that indicated lack of enough energy for daily activities and unsatisfied with capacity to work had poorer QoL.

### 3.5. Association between Social Relationships and Perceived QoL

Social relationships were significantly associated with QoL. The determinants were satisfaction with personal relationships (*X*^2^ = 31.353; *p* < 0.001) and feelings of loneliness (*X*^2^ = 21.083; *p* < 0.001). Furthermore, majority (93%) of women were dissatisfied with their personal relationships, and 94% that had felt loneliness had poorer QoL.

### 3.6. Association between Environmental Determinants and Perceived QoL

Environmental determinants were significantly associated with QOL, and these included opportunity for leisure (*X*^2^ = 49.854; *p* < 0.001), felt safe in daily life (*X*^2^ = 52.161; *p* < 0.0010), health of physical environment (*X*^2^ = 38.945; *p* < 0.001), satisfaction with condition of living place (*X*^2^ = 38.945; *p* < 0.001), availability of enough money to meet daily needs (*X*^2^ = 41.236; *p* < 0.001), and access to information required in day-to-day life (*X*^2^ = 13.935; *p*=0.030). Also, majority of women indicated that lack of opportunity for leisure activities (96%), felt unsafe in daily life (94%), limited access to information (93%), unhealthy physical environment (100%), and lack enough money to meet needs (100%) had poorer QoL.

### 3.7. Association between Psychological Determinants and Perceived QoL

Psychological determinants associated with QoL included negative feelings (*X*^2^ = 20.336; *p*=0.002), accepting bodily appearance (*X*^2^ = 15.527; *p*=0.004), self-confidence (*X*^2^ = 46.800; *p* < 0.001), enjoying life (*X*^2^ = 45.131; *p* < 0.001), meaningful life (*X*^2^ = 27.839; *p* < 0.001), ability to concentrate (*X*^2^ = 34.869; *p* < 0.001), and self-satisfaction (*X*^2^ = 18.171; *p* < 0.001). Majority of women (86%) reportedly had negative feelings, 88.9% were yet to accept their bodily appearance, 100% had no self-confidence, 100% perceived life as meaningless, 96% were unable to concentrate, and 90% were not satisfied with themselves, and had poorer QoL.

At multivariate level, ordinal logistic regression model was used to establish the association between the independent variables which were significantly associated with QoL. As indicated in Table 3, self-confidence and formal education were significant predictors of QoL. On the other hand, women who did not experience difficulties in mobility (OR = 19.144; CI = 0.149–2456.770) and those satisfied with their personal relationships (OR = 5.785; CI = 0.447–74.824) were more likely to have good QoL.

### 3.8. Satisfaction with Life

According to Table 4, among the four domains of WHOQoL, the highest mean satisfaction rating was found for (physical health, Mean = 8.460), implying good activities of daily living, less dependence on medication, enough energy and mobility, less pain and discomfort, sufficient sleep and rest, and good work capacity. Moreover, the lowest mean score was shown for (environmental support, Mean = 5.951), indicating limited financial resources, opportunities for acquiring new information and skills, and leisure activities.

### 3.9. Living with OF

The study revealed that women reported a change in way of life resulting from constant leakage of urine. The physical experience according to the women was unbearable; 7 out of 9 women reported experiencing sores and burns especially in genital areas and on the thighs and 2 out of 9 women experienced uncontrolled leakage of feces. Consequently, the women devised means to avoid being noticed and used locally made pads which also required to be changed often. This is physically exhausting as illustrated in the following quotes:

“I have an excessive and uncontrollable leakage of urine and a painful rash around my genital area. I wake up several times during the night to change my bed sheets. I thought that if I reduced taking water, the urine would stop. However, this did not work as the urine started to have a very bad odor.” **FGD 3**.

*“I cannot control my faeces. I no longer leave my home, everywhere I used to go people pointed figures at me and said there comes, “the smelly woman,” I have tried padding myself with lots of clothes but they get soaked in short time. I spend most of times at home washing these clothes and am afraid of meeting people because they will immediately notice my problem*.” **FGD 4**.

“I did not know this condition and I was advised to take some herbal medicine. Which I did for several days. However, I was not healed. This leaking continued until today.” **FGD 3**.

### 3.10. Stigmatization and Isolation from Community

Majority of the women experienced stigmatization and isolation arising from leakage of urine or feces. They were discriminated by husbands, relatives, and friends upon realizing that they were uncontrollably leaking urine. The women also reported to have isolated themselves from peers as they could not afford interactions for long.

“I stopped going to Church because my community thinks am cursed by a God. I have no friends because all people avoid me due to the bad smell.” **FGD 1**

“I cannot travel long distances because I was stigmatized from people especially the boda-boda men and taxi operators due to bad smell of the urine.” **FGD 6**

“Am not employed anymore. I stopped working at the saloon because my employer complained of my bad smell. She said customers were no longer coming to her salon because of me.” **FGD 4**

“My husband no longer shares a bed with me and does not take me for social events anymore because of my condition.” **FGD 7**

### 3.11. Sexual and Martial Life

More than half of the women reported to have lost their child following fistula. Their husbands blame them for the loss. 6 out of 9 women reported their husband abandoned them for another woman as result of fistula. Most of the women felt their marital and sexual lives were no longer joyful.

“I hide my condition from my husband for one month but when he discovered it. He could not bear the smell and constant wetness of our bed so he left me for another woman.” **FGD 1**

“My baby died during delivery. Am afraid to get pregnant again because I fear my baby will die.” **FGD 7**

“Am afraid to get intimate with any man because of my condition. I fear that once he discovers my condition he will tell everyman in the community.” **FGD 6**

## 4. Discussion

### 4.1. Sociodemographic Characteristics of Women with Obstetric Fistula

In this study, more than half of the women were divorced or separated. This finding is in agreement with a previous study where 57% of women were divorced [12]. This is due to the fact that these women either isolated themselves or were isolated by society, including by close relatives and their husbands [12]. In addition, these women reported that their sexual lives were no longer enjoyable and felt a loss of their marital and sexual rights. With regard to age at first pregnancy, a vast majority of women had their first pregnancy less than 20 years. This finding is similar to a previous report which reported that more than half (55%) of the women had their first marriage between the ages of 15–20 years [17]. From this study, it was revealed that young age at marriage among women with OF was 15.5 years. This is probably because child marriages lead to early pregnancies when the body is undeveloped for delivery of baby consequently resulting in fistula which negatively affects QoL [20]. Furthermore, the study established that more than half of the women had attended primary level education. The odds of good QoL were 9.9 times more likely among those who had formal education compared to those who did not. This finding is similar to an earlier report where majority of the women were illiterate [21]. This is probably because women who are educated are more informed about prevention of fistula and have more opportunities to participate in income generating activities to improve their QoL [5, 12, 21].

The study established a significant association between level of education and QoL with majority of women with no formal education having poorer QoL. This is probably a result of limited knowledge and skills to create an enabling environment for better QoL. In addition, employment status was significantly associated with QoL with majority of women not employed having poorer QoL as a result of limited opportunities for financial empowerment. This is consistent with other studies [12, 13, 16, 19].

### 4.2. Physiological Determinants of QOL among Women with Obstetric Fistula

In this study, experience of physical pain and difficulties in mobility were significantly associated with poor QoL. This is probably due to physical pain and mobility difficulties limiting a woman's ability to perform daily activities that contribute to her wellbeing. This is in line with other studies [17, 22]. Also, women complained of an array of physical consequences associated with the fistula, including rashes, boils, and ulcers that developed around the vulva and thighs, causing itching and severe burning and leading to vaginal infections. Also, reports indicate that fistula patients experience physical changes, such as weight loss, loss of bodily control, pain around the pelvis, physical exhaustion, and an inability to walk or work [13, 23]. The study also established that lack of energy for everyday life was associated with poor QoL. This is attributed to physical exhaustion and loss of independence resulting from uncontrollable leakage of urine. There was an association between satisfaction with sleep pattern and QoL with majority of women reporting dissatisfaction with sleep having low QoL. This is due to continuous leakage of urine accompanied by its bad odor even during the night when women had to awake from their sleep to change beddings. This is consistent with earlier reports [23, 24]. The study established a significant association between capacity for work and QoL, with majority of the respondents reporting dissatisfaction with work having low QoL. This is because the leakage of urine has several repercussions on the woman's QoL among inability to engage in meaningful employment thereby impacting their QOL as already established [12, 23, 24].

### 4.3. Psychological Determinants of QOL among Women with Obstetric Fistula

In this study, majority of women reported having negative feelings such as blue mood, despair, anxiety, and depression which was associated with poor quality of life. This is probably due to loss of hope for cure as a result of this condition. This correlates to an earlier report in which a feeling of depression was reported by 92% of untreated women and suicidal ideation was experienced in more than half of the women with feelings of depression [25]. In addition, this suggested that women with obstetric fistula had high prevalence of depressive symptoms and asserts that interventions that are tailored to increase self-efficacy may improve depression and quality of life among obstetric fistula clients [26, 27]. Additionally, the study established a significant association between acceptance of bodily appearance and QoL. Women who found it difficult to accept their bodily appearance exhibited poor QoL. This is due to the associated diseases among dermatitis that lower their QoL. This is consistent with other studies [28, 29].

The study also established a significant association between self-confidence and QoL. Women who lacked self-confidence exhibited poor QoL. This is due to the bad smell of urine; women isolated themselves from the community and also failed to seek treatment hence impacting QoL, similar to previous reports [30, 31].

### 4.4. Social Relationships among Women with Obstetric Fistula

In this study, FGD established that the women devised means to avoid being noticed and padded themselves with locally made pads which also required to be changed often. This was physically exhausting. Majority of the women experienced stigmatization and isolation arising from leakage of urine or feces. They were discriminated by husbands, relatives, and friends upon realizing that they were uncontrollably leaking urine. This is consistent with a previous report where women were socially isolated and rejected, stigmatized, and depressed and there was no joy in their sexual life [12]. In attempt to fight the stress that results from leakage, the women with fistula adopted both problem- and emotion-focused coping strategies. As a result, they had to think about their every movement; when sitting, for instance, they had to consider whether they had enough protection to avoid wetting the bed or chair. The study revealed significant relationship between QoL and social relationships. Majority of women who were dissatisfied with their personal relationships and felt loneliness had poorer QoL. These women faced stigmatization and isolation from their community, relatives, and husbands that abandoned them due the condition hence exhibiting low QoL. An earlier report showed that these women are often considered social outcastes and labelled as shameful [12]. Also, majority of the women were very dissatisfied with their sexual life. This is due to the genital sores and inflammation that made sex painful. These findings are consistent with a previous report where 25% of women reported that the urinary incontinence contributed to a decrease in sexual life [32]. Sexual dissatisfaction in women resulted in a decreased personal and marital quality of life. This study established that majority of the women's QoL was affected by obstetric fistula due to its severe consequences. These findings demonstrate consensus with other studies where 66% of women reported that their quality of life was affected by urinary incontinence [33, 34].

## 5. Conclusion

Our findings demonstrate a consensus regarding the influence of education level, employment status, experience of physical pain in daily life, social relationships, and self-confidence on the quality of life of obstetric patients across countries. Based on these, interventions point to a need for a holistic treatment approach to address the consequences of obstetric fistula to realize improved quality of life of patients. In this regard, policy interventions ought to focus on adequate community sensitization and awareness about obstetric fistula.

## Figures and Tables

**Table 1:** Sociodemographic factors of women with obstetric fistula.

Background characteristics	Frequency (*n* = 63)	Percentage (%)
Age		
Below 20	9	14
20–29	34	54
Above 29	20	32

Education level		
None	20	32
Primary	34	54
Secondary	9	14

Employment status		
No	44	70
Yes	19	30

Marital status before fistula		
Married but not living with partner	10	16
Married living with partner	41	65
Separated	3	5
Single (never married)	8	13
Widowed	1	2

Marital status after fistula		
Married but not living with partner	9	14
Married living with partner	8	13
Separated	35	56
Single (never married)	8	13
Widowed	3	5

Age at first pregnancy		
Below 20	45	71
20–29	13	21
Above 29	5	8

Incontinent		
RVF	8	13
VVF	51	81
VVF + RVF	4	6

Duration with fistula		
1_12 months	29	46
1_2 years	20	32
2_5 years	14	22

Outcome of delivery preceding fistula		
Early neonatal death	10	16
Live birth	9	14
Still birth	44	70

Source: primary data from women.

**Table 2:** Association between patients' sociodemographic factors and perceived QOL.

Variable	Observation (*n* = 63)	Perceived quality of life				*p* value
		Poor (%)	Fair (%)	Good (%)	*χ* ^2^	
Age						
Below 20	9	100.0	0.0	0.0	4.541	0.338
20–29	34	91.2	5.9	2.9		
Above 29	20	75.0	15.0	10.0		

Education level						
None	20	95.0	5.0	0.0	22.923	<0.001
Primary	34	94.1	5.9	0.0		
Secondary	9	44.4	22.2	33.3		

Employment status						
No	44	100.0	0.0	0.0	21.221	<0.001
Yes	19	57.9	26.3	15.8		

Marital status after fistula						
Married but not living with partner	9	77.8	11.1	11.1	15.261	0.054
Married living with partner	8	50.0	37.5	12.5		
Separated	35	94.3	2.9	2.9		
Single (never married)	8	100.0	0.0	0.0		
Widowed	3	100.0	0.0	0.0		

Age at first pregnancy						
Below 20	45	91.1	2.2	6.7	10.949	0.027
20–29	13	84.6	15.4	0.0		
Above 29	5	60.0	40.0	0.0		

Incontinent						
RVF/VVF + RVF	12	100.0	0.0	0.0	2.156	0.340
VVF	51	84.3	9.8	5.9		

Duration with fistula						
Less than 1 year	29	86.2	10.3	3.4	0.595	0.743
More than 1 year	34	88.2	5.9	5.9		

Outcome of delivery preceding fistula						
Lost child	54	90.7	9.3	0.0	19.345	<0.001
Live birth	9	66.7	0.0	33.3		

**Table 3:** Multivariate analysis of the determinants associated with QOL.

Item	Intercept	*p* value	Odds ratio	95% CI for odds ratio	
				Lower	Upper
Formal education	2.298	0.043	9.9497	1.075	92.048
Personal relationships	1.755	0.179	5.785	0.447	74.824
Self-confidence	3.476	0.014	32.320	2.019	517.467
No difficulties in mobility	2.952	0.233	19.144	0.149	2456.770

**Table 4:** Satisfaction with life.

	*N*	Mean	Std. deviation	Std. error	95% confidence interval for mean		Minimum	Maximum
					Lower bound	Upper bound		
Physical health	63	8.460	1.9905	.2508	7.959	8.962	6.0	12.0
Social relationship	63	6.063	1.3425	.1691	5.725	6.402	5.0	10.0
Environment	63	5.951	2.1667	.2730	5.405	6.496	4.0	12.0
Psychological	63	6.690	1.5974	.2013	6.288	7.093	4.5	11.0

## Data Availability

The authors did not obtain consent to share data obtained; however, the datasets used and/or analyzed during the current study are available from the corresponding author on reasonable request.

## Abbreviations

FGD: Focus group discussion
OF: Obstetric fistula
QoL: Quality of life.
